# Integrating HEC-RAS, GIS, and LISREL for assessing and enhancing urban building resilience against flood threats: Comprehensive model and analysis

**DOI:** 10.1016/j.heliyon.2024.e39463

**Published:** 2024-10-16

**Authors:** Faraz Estelaji, Rahim Zahedi, Arash Gitifar, Alireza Naseri, Mohammad Hossein Yari, Bita Rouhi Asl, Bita Abedi

**Affiliations:** a Department of Construction Engineering and Management, Faculty of Civil Engineering, Khajeh Nasir Toosi University, Tehran, Iran; b Department of Energy Governance, University of Tehran, Tehran, Iran; c Department of Environmental System Engineering, University of Regina, Saskatchewan, Canada; d Department of Road and Transport Engineering, Faculty of Civil and Environment Engineering, Amirkabir University of Technology, Tehran, Iran; e Faculty of Civil Engineering, Sharif University of Technology, Tehran, Iran; f Department of Environmental Management, Faculty of Marine Science and Technology, Azad University, Tehran, Iran; g Department of Remote Sensing, Faculty of Geodesy and Geomatics, Khajeh Nasir Toosi University, Tehran, Iran

**Keywords:** *Flood simulation*, *LISREL*, *HEC-RAS*, *GIS*, *Resilience*, *Sustainability*

## Abstract

Floods pose significant threats to urban areas, resulting in substantial human and financial losses annually. The vulnerability of key urban centers to these risks diminishes their efficiency, leading to public dissatisfaction and service deficiencies. Recognizing and enhancing the resilience of essential buildings becomes crucial in mitigating these challenges. This study employs a comprehensive approach to achieve a resilience model for critical buildings facing floods. The research process involves the identification of city assets based on leveling criteria, utilizing GIS technology for spatial mapping. HECRAS software aids in river flow modeling, identifying areas lacking flood-carrying capacity. By overlaying vulnerable gravity centers with flood-prone regions, building resilience components are computed through structural factor analysis and LISREL modeling. The study identifies ten key criteria. Further analysis includes resilience modeling using TOPSIS and AHP methods. The positive ideal and negative ideal solutions are determined, resulting in the grading of building resilience. Notably, the balance redundancy index with cascading potential effects attains the highest positive ideal value at 0.257, while the resistance to a level of stress index achieves the lowest negative ideal value at 0.02. This comprehensive approach and modeling contribute to the understanding and enhancement of urban building resilience in the face of flood threats.

## 1 Introduction

The contemporary urban landscape grapples with a paramount challenge; environmental hazards, a pivotal concern for urban communities [1]. Effectively identifying, managing, and controlling these hazards is imperative [2]. Among these perils, floods emerge as particularly destructive natural phenomena, causing substantial human and financial losses annually, with their intensity on the risen [3]. Consequently, the scrutiny of vulnerability and resilience to floods becomes paramount [4]. It is crucial to assess and fortify key urban centers to enhance their resilience [5]. Recognizing and evaluating the vulnerability of these centers to various risks and threats is a prerequisite for effective resilience [6].

The assessment of resilience against urban flooding necessitates a comprehensive understanding of the drainage system's ability to withstand floods and recover [7]. However, despite the growing demand for heightened resilience, success has often proven elusive [8]. The inherent challenges of advancing the resilience of intricate and adaptable systems, such as cities, have been largely underestimated [9]. Therefore, the development and application of approaches to bolster resilience are critical, given the intricate nature of urban systems [10]. A city's sustainability hinges on its capacity to mitigate vulnerability to hazards and respond innovatively to changes [11]. Given the perpetual risks faced by cities, the crux of urban resilience lies in the system's ability to protect itself or swiftly return to an ideal state [12]. Urban resilience, as a concept, entails the adeptness to respond to diverse and plausible perspectives on risks or a cluster of risks [13].

In addressing urban floods, considerable efforts have been invested in flood assessment methods and frameworks [14]. These methods typically employ multi-criteria indicators to evaluate various facets of flood resilience [15]. Despite widespread implementation of flood control infrastructure globally, modern cities remain susceptible to flood hazards [16]. Consequently, fortifying the resilience of urban buildings becomes an indispensable facet of overall urban resilience [17]. This discourse delves into pertinent research endeavors on urban resilience in the face of disasters such as floods, highlighting some of the most pivotal studies in this domain [18].

In 2023, Wang et al. [19] employed the metric base cell network method to assess the resilience of the urban basin within the Dalian region of China. This urban basin, divided into 31 sub-basins, underwent evaluation in the context of flood response. The CADDIES model was utilized for flood simulation. The study identified vulnerable basins and formulated strategies to enhance the city's resilience against floods. A comparative analysis with existing metrics revealed that the new metric provides a comprehensive reflection of system performance changes. Consequently, tailored strategies for increasing resilience in each urban area were devised.

Salimi et al. [20], also in 2020, investigated the resilience of urban buildings and the impact of mass production in confronting climatic risks, particularly flooding, within the metropolitan area of Mexico. Their work focused on studying the resilience of buildings in the Mexican capital over the past decade amid multiple flood events. The findings underscored that building resilience is a complex, ongoing process influenced by social, economic, and institutional factors.

In a 2020 study by Mohammadzadeh et al. [21], the authors emphasized the importance of identifying the extent and severity of potential damages when addressing threats. Their research specifically examined the flood threat to the intra-city rail transportation infrastructure in Mashhad. Results indicated that the sub-section of control equipment presented the highest risk priority number at 43.06, highlighting its heightened vulnerability compared to other sections to flood threats.

Klijn et al. [22], in their article assert that flood risk management constitutes a comprehensive approach encompassing various indicators. Numerous assessment methods exist for evaluating flood risk in river watersheds, and while effective and robust, they are inherently complex. Flood risk assessment plays a pivotal role in furnishing valuable insights for flood risk management, particularly in evaluating vulnerability and exposure to risk. Various models, such as DSS, RF SVM, RFM, ANN, and GA, have been employed in flood risk assessment within river basins. These models aim to investigate the influential indicators in flood risk and ascertain the degree of importance attributed to these indicators in different watershed areas.

Yaryan et al. [23] employed fuzzy logic to assess the vulnerability of buildings and residential units based on various criteria. The evaluation involved assigning degrees of vulnerability to each criterion, and the cumulative assessment of all criteria yielded the final percentage of vulnerability. Lamond et al. [24] focused their work on urban rivers and resilience against flood chaos, particularly in the planning of the Ken River. Drawing insights from four successful global projects, the research extracted principles and solutions for enhancing resilience against river floods. Key principles included time, trial, threshold, learning, and diversity, with a specific emphasis on stratification in river banks and wetlands as a solution to address riverbank-related challenges. The integration of various sciences in this matter emphasized the need for multidisciplinary interaction and planning across different disciplines. This comprehensive and multidisciplinary planning, rooted in flood resilience thinking, serves as a potential model for other urban rivers vulnerable to flood chaos.

Upon examining various databases, it was revealed that extensive research has been conducted in the realm of urban resilience, covering concepts, dimensions, and measurements across different natural hazards. However, the present research focuses specifically on the investigation of natural flood crises as a threat to crucial, sensitive, and vital buildings. In summary, the research gaps identified in the assessing and enhancing urban building resilience against flood threats are as follows; Building risk analysis based on flood depth and adaptation measures for buildings at risk. Hence, the objectives chosen for this study are (a) to develop flood inundation maps and identify the submerged areas for RCPs 6.0 and 8.5, using HEC-RAS 2D, (b) to compute building risk analysis based on flood depth, and (c) to formulate suitable adaptive measures and assess their effectiveness for attenuation of building risk in the urban catchment. The applied nature of this research aims to propose solutions for building resilience, especially those structures posing significant threats to the provision of information, communication, services, and resources in the city during instances of damage and destruction.

## 2 Methodology

The majority of existing research on floods predominantly focuses on vulnerabilities arising from flood events. These studies typically consider both vulnerability and resilience as interconnected facets. However, it is noteworthy that a building may not only be vulnerable to floods but also lack the resilience necessary to withstand them effectively. This could manifest in various ways, such as the functional area being affected due to blocked roads or the building being susceptible to other threats, undermining its overall performance and efficiency.

This research diverges by framing building resilience as an inherent characteristic within complex systems, particularly key buildings. The study aims to validate the critical components contributing to resilience. A distinctive aspect of this research lies in its process-oriented approach. Unlike conventional studies that predominantly assess the structure and construction of buildings, this approach delves into the performance-based resilience of focal points. Moreover, it doesn't limit examination solely to a specific risk or threat but broadens the scope to consider the resilience of centers of gravity.

When comparing vulnerability indicators extracted from FEMA guidelines with resilience indicators calculated in this study, a key revelation emerges [25]. The process-oriented approach demonstrates that the resilience of focal points extends beyond mere structural aspects, encompassing overall performance [26]. On the other hand, the result-oriented approach emphasizes that reducing vulnerability in a building's performance is an integral component of its overall resilience [27].

Unlike analogous research endeavors focusing on building resilience, this study endeavors to identify and rank the components with the least and most impact on resilience. This ranking forms the basis for designing an operational plan that prioritizes indicators for the development of resilient buildings. This research encompasses a multi-stage data analysis approach to develop a resilience model for critical buildings against floods. The sequential steps include.

1. Identification of Critical Assets:

The initial phase involves identifying pivotal assets from the perspective of non-operational defense [28].

2. River Flow Modeling:

Utilizing HECRAS software, the research incorporates river flow modeling to comprehensively understand and simulate the dynamics of water flow in the river [29].

3. Alignment of Assets and River Modeling Results:

A critical step involves matching the identified assets with the modeling results obtained from the river flow simulations across various return periods [30].

4. Assessment of Flood-Affected Assets:

Quantifying the impact of floods on assets, this stage involves counting the number of assets significantly affected by flood events [31].

5. Resilience Component Modeling:

The study employs structural equation modeling through LISREL software to determine and model the various components contributing to the resilience of the building [32].

6. Component Ranking:

Employing the combined AHP-TOPSIS method, the identified components are systematically counted and ranked based on their significance within the resilience model [33].

7 Ranking of Flood-Affected Key Buildings:

Utilizing the AHP-TOPSIS combined method, the research proceeds to rank key buildings in terms of their vulnerability to floods, providing a comprehensive assessment of the impact on critical structures [34].

This methodological framework ensures a thorough analysis, integrating various techniques and software tools to develop a robust resilience model for essential buildings facing the challenges posed by floods.

### 2.1 Study and verification of location and study area

**Fig. 1:**
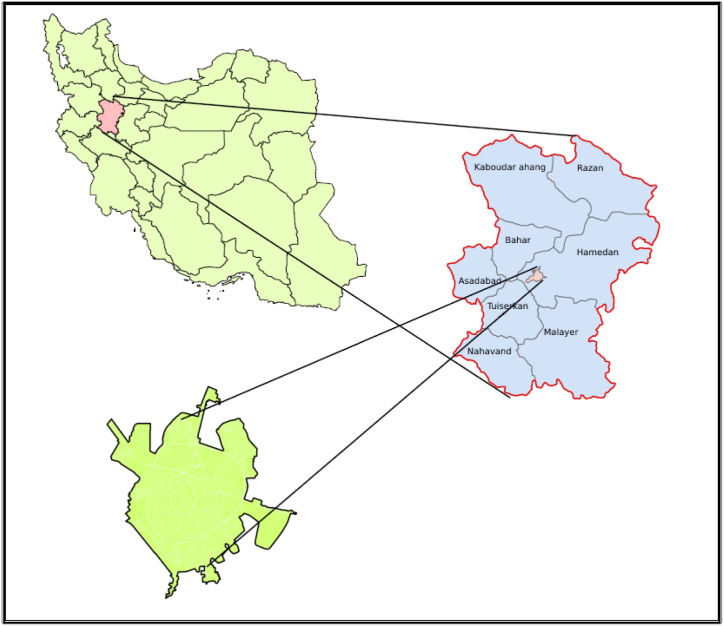
Geographical location of Hamedan city.

The city of Hamadan is positioned within the geographical coordinates of 28–48° longitude and 33° 48 min to 45° 34 min latitude, with an elevation of 1870 m above sea level [35]. Historically, Hamadan has served as a crucial hub for communication routes from western cities to the central regions of Iran [36]. Situated in the midst of a plain linked to Ganjnameh valley in the west, the city is traversed by the Abbas Abad River, originating from Alvand Mountain [37]. Refer to Fig. 1 for a visual representation of the geographical location of Hamadan city.

The study's scope encompasses the detailed city development plan, covering an area of approximately 7200 ha (72 square kilometers). It's noteworthy that this defined area aligns with both the Hamadan sewage network plan and the surface water collection and flood management plan. These plans, formulated by the criteria set forth in the same area, contribute to the comprehensive nature of the study [38]. For instance, meteorological and hydrological investigations were conducted over an approximately 100-square-kilometer zone to analyze and ascertain the flooding patterns of rivers and canals entering the city of Hamadan.

### 2.2 Identification of important assets

Recognizing and prioritizing city assets is a foundational step crucial to the subsequent analyses in this research. The accuracy of asset identification and prioritization during this phase significantly influences the effectiveness of subsequent stages of the study [39]. The primary objective of this stage is to identify assets that, in the event of damage and destruction, pose a substantial threat to the provision of information, communications, services, and resources in Hamadan city.

To achieve this goal, a consolidated method has been employed, integrating the perspectives of managers from various fields related to urban affairs, drawing on reliable scientific sources, and leveraging existing experiences. Employing this consolidated approach, the assets of Hamadan city are categorized into four main groups.

1. Physical Assets:

This category includes three subgroups: capital, facilities, and structures [40].

2. Human Resource Assets:

Recognized as the most vital asset, the human resource category plays a central role in the overall asset classification [41].

3. Information Exchange or Cyber Assets:

Encompassing a range of networks such as computers, software, internet infrastructure, satellites, and databases, these assets are designed to facilitate the transfer of information and data [42].

4. Spiritual Assets:

Comprising lasting symbols, national honor, and elements of independence, spiritual assets hold significant value for every country, as outlined by Ref. [43].

This comprehensive classification serves as the foundation for the subsequent phases of the research, providing a systematic framework for analyzing and enhancing the resilience of Hamadan city against potential threats and challenges.

### 2.3 River flow modeling using HEC-RAS software

This section of the research focuses on the examination and study of assets susceptible to flood threats, specifically delving into the study of rivers and their flow modeling. Hydrological studies in this context aim to determine the design flood of each river [44]. To ensure consistency and unity in the obtained flood data, an integrated modeling approach is adopted, encompassing both suburban and inner-city catchment areas of the rivers.

Hydraulic parameters along the rivers and at different stages are crucial for understanding flood dynamics [45]. Therefore, the selection of reliable software capable of solving equations for permanent or non-permanent variable flows is imperative, given the high volume of operations and the critical nature of the information. Introduced around 1990 for modeling steady flows in one dimension, HEC-2 software demonstrated the ability to model cross structures such as bridges and culverts [46]. Its latest iteration, HEC-RAS, has evolved to offer extensive capabilities. This includes the solution for unsteady flows, mixed flow calculations, quasi-two-dimensional velocity distribution in sections, output to the GIS environment for flood plains, and various other features. The latest version positions HEC-RAS as a professional and reliable software tool [47].

Given these advanced features, HEC-RAS software has been employed in this research for hydraulic modeling of rivers. Its capabilities contribute significantly to the accurate analysis of river dynamics, enhancing the understanding of potential flood impacts on assets within the study area.

### 2.4 Adaptation of assets and hydraulic modeling of rivers

The integration of asset adaptation and hydraulic modeling of rivers employs overlapping functions within Geographic Information System (GIS), generating maps to address specific research questions [48]. Various overlay methods exist, and the choice depends on the type of data used and the overarching purpose of the overlay [49]. In this research, the selected method is feature overlay. This approach yields new complexities that, while retaining the nature of existing complexities, introduce additional features pertinent to the research context. The feature overlay method facilitates the synthesis of data, allowing for a comprehensive analysis that enhances the understanding of asset vulnerability and river dynamics in the study area.

### 2.5 Determining building resilience components

The Resilience Index, an evaluation method developed by Argonne National Laboratory, serves as a valuable tool for comparing the resilience levels of critical infrastructures [50]. This method aids in prioritizing limited resources for enhancing resilience [51]. The indicators utilized in this index rely on subjective evaluations from experts, focusing on three key characteristics of resilience: Strength, Resourcefulness, and Recovery.

1. Strength [52]:

Encompasses the ability to maintain critical operations and performance in the face of a crisis.

2. Resourcefulness [53]:

Involves the ability to prepare, respond, and effectively manage a crisis or disturbance.

3. Recovery [54]:

Signifies the ability to promptly and efficiently return or restore normal operations.

Factors such as redundancy, diversity, connection, and cascading effects, often overlooked, play a pivotal role [55]. Table 1 outlines the primary dimensions of resilient systems identified in past RAMSES workshops, forming the basis of questionnaires in this research. The rating criteria for building resilience and the resilience model are derived from these dimensions.

**Table 1:** Questionnaire dimensions and items.

Row	Dimensions	Dimensions
Adaptability flexibility - QM	Change while maintaining or improving performance	Timely response to changing conditions
	Rapid evolution and change	Open design and flexible structures
	Rapid adoption of alternative strategies	
Failure safety feedback connection - EL	shock absorption	failure without cascading effects (domino effect)
	Absorbing the cumulative effects of slow-onset challenges	Pairwise analysis of human-technology system
	Avoid catastrophic failure if threshold is exceeded	Identify the blocking effects and possible contradictions with reduction
	Gradual rather than sudden failure	Identifying synergies with other city policies, estimating added value
Dependence on environmental ecosystems - GI	Flood control	Bio-climatic design and management
Variety of JD	Spatial diversity	Functional diversity - multiple ways of dealing with a particular need
	Key assets and tasks that are physically distributed and not all affected by a specific event at any time.	Equilibrium variation with potential cascading effects
Learning _ Memory _ Prediction GM	Learning from past experiences and failures	Collect, store, and share experience
	Use information and experience to create new adaptations	Construction based on long-term value and history of the city
	Avoid repeating past mistakes	Integrating resilience into long-term development scenarios
GT performance	Performance capacity	Self-sufficiency - reducing external dependence
	System quality	It performs better than other buildings
HF response speed	In taking casualties, including death and illness	Restore the structure
	Reorganization	Establishing public order
	Maintaining performance and restoring it	Prevent future disruption
DS segmentation redundancy	Replacement of systems or systems agents	Replacing components with modular parts
	Buffer from external shocks or changes in demand	Balance redundancy with potential cascading effects
OR strategy	Identifying and predicting problems	re-evaluation
	Prioritize	Integrating resilience into work processes and administration
	Resource mobilization, visualization, planning, collaboration and action	Getting cooperation from citizens
IP strength	Resistance to a level of stress	Capacities that guarantee adequate margins
	Without degradation and loss of performance	

In the analysis phase, the resilience items and dimensions undergo thorough investigation and validation. Utilizing chi-square and factor analysis, their impact on building resilience in flood scenarios is extracted. This rigorous analysis ensures the reliability and effectiveness of the identified resilience components, contributing to the development of a robust model for building resilience against floods.

### 2.6 Utilizing LISREL modeling software for building resilience models

LISREL, developed by Scientific Software International, stands as a specialized software designed for the estimation and testing of structural equation models [56]. This software, leveraging measured correlation and covariance data, possesses the capability to estimate or infer values such as factor loadings, variances, and errors associated with underlying variables [57]. LISREL is a versatile tool, enabling researchers to conduct exploratory factor analysis, second-order factor analysis, confirmatory factor analysis, and path analysis – the latter involving cause-and-effect modeling with latent variables [58].

One particularly valuable technique within the realm of structural equation modeling is Covariance Structural Analysis, also known as structural linear relationships [59]. This approach allows for a comprehensive examination of relationships among variables, offering insights into the complex interplay of factors influencing building resilience [60]. The utilization of LISREL in this research facilitates a robust and nuanced analysis, providing a sophisticated model for understanding and enhancing building resilience against flood-related challenges.

### 2.7 Ranking resilience indicators of key buildings using the combined AHP-TOPSIS method

This research employs the Analytic Hierarchy Process (AHP) to rank building resilience against floods. Achieving a comprehensive ranking necessitates the identification and analysis of multiple criteria and indicators. To address this, the research initiates by determining and validating criteria and sub-criteria crucial for evaluating building resilience against floods.

Given the diverse impacts of these indicators, a rigorous approach is employed. Using the AHP technique, 50 experienced experts in the field of resilience and passive defense engage in a pairwise comparison of these components. This expert-driven assessment allows for the calculation of the final weight for each criterion and sub-criterion [61]. This weightage forms the foundation for subsequent analyses and rankings, ensuring a robust and informed evaluation of building resilience [62]. The AHP method serves as a crucial component in establishing a comprehensive framework for the subsequent application of the TOPSIS method in ranking resilience indicators for key buildings.

## 3 Results and discussion

**Fig. 2:**
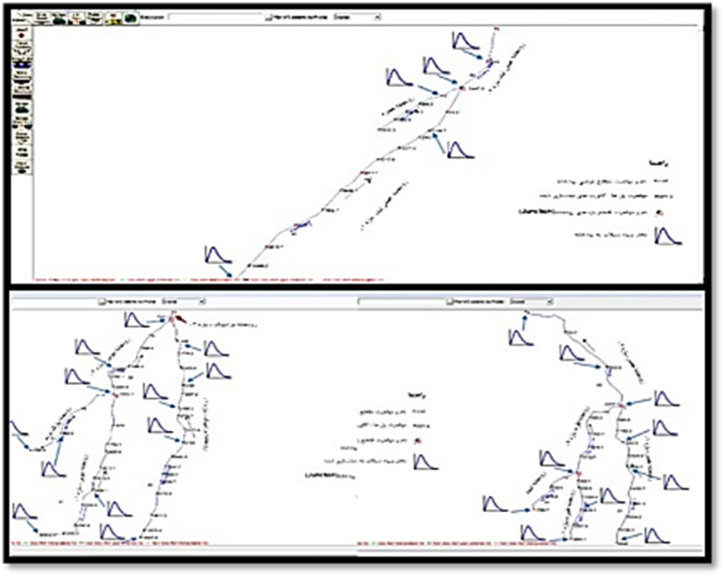
River flow Modelling.

The examination of longitudinal profiles reveals that in certain segments of these rivers, the existing conditions are unsatisfactory, leading to the discharge of designed floodwaters beyond the channel. Consequently, roads experience inundation. The inadequate capacity of river sections, coupled with the inappropriate dimensions of bridges and culverts, constitutes the factors contributing to the incapacity of Hamadan's rivers to safely divert floodwaters away from the city as shown in Fig. 2.

**Fig. 3:**
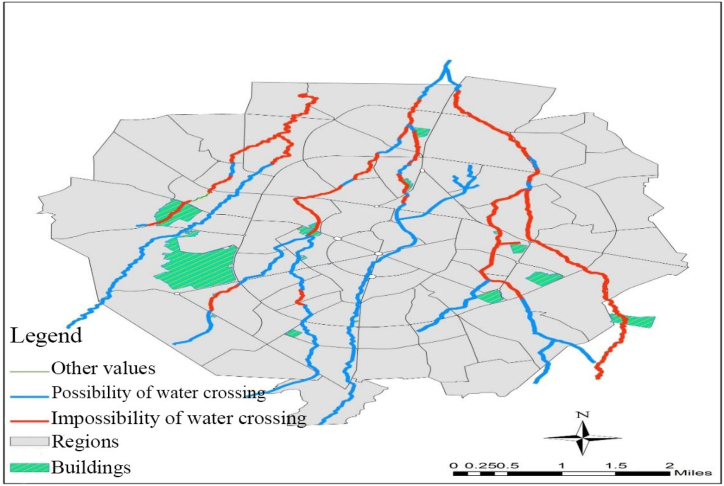
Adaptation of the assets layer of Hamedan city and sections of the city's rivers flood permeability.

The asset layers of Hamedan city and its rivers were aligned within the GIS platform. Through this alignment, five notable buildings were found as vulnerable focal points in Hamedan. The resulting map is depicted in Fig. 3.

In the analysis section of the research findings, the initial focus was on examining the items and dimensions (Table 1). Subsequently, the impact of each dimension and item on building resilience in floods was assessed using the Chi-square test. Following this, the influencing factors and the magnitude of their effects within the dimensions were measured using factor analysis.

After determining the structural resilience of the building in the flood, compatibility and flexibility in the subsequent sections were assessed for each of the defined components and criteria. The status of each defined component for both dimension and criteria was analyzed using the chi-square (χ^2^) single-variable test, owing to the non-normality of the data (Table 2).

**Table 2:** Attitude of adaptability - flexibility.

object	Frequency distribution - percentage					Average	Chi-square value	Significance level (Sig)
	very low	low	moderate	high	very high			
**Change while maintaining or improving performance**	3.30	8.50	9.8	35.00	43.40	4.07	235.995	0.000
**Rapid evolution and change**	7.40	10.90	19.1	16.9	45.60	3.83	166.268	0.000
**Rapid adoption of alternative strategies**	3.00	4.60	3.8	36.9	51.90	4.30	379.246	0.000
**Timely response to changing conditions**	3.90	4.60	8.5	14.8	63.80	4.17	429.383	0.000
**Open design and flexible structures**	3.60	3.80	10.9	18.00	63.70	4.34	462.005	0.000

The obtained results regarding the structural resilience of the building under flood conditions for each of the connection components are outlined in Table 3. As indicated in the table, considering the single-variable chi-square (χ^2^) values and the significance level smaller than 0.05, there is a statistically significant difference in all studied connection components between the observed frequencies and the expected frequencies. Since the highest frequencies are observed in all components for the moderate, high, and very high-rise categories, it can be concluded that the structural resilience of the building under flood conditions has a significant impact on the safety failure of each feedback connection component.

**Table 3:** Attitude of connection-Feedback-Safety-Failure.

object	Frequency distribution - percentage					Average	Chi-square value	Significance level (Sig)
	very low	low	moderate	high	very high			
**shock absorption**	9.30	4.40	6.60	15.30	64.50	4.21	464.87	0.000
**absorbing the cumulative effects of slow-onset challenges)**	13.4	27.00	12.60	24.60	22.50	3.16	32.12	0.000
**Avoid catastrophic failure if threshold is exceeded**	7.9	9.30	3.00	13.40	66.40	4.21	502.42	0.000
**Gradual rather than sudden failure**	3	1.40	13.40	17.80	71.60	4.54	638.70	0.000
**failure without cascading effects (domino effect)**	20.2	13.70	17.80	19.10	20.20	3.05	15.91	0.000
**Pairwise analysis of human-technology system**	14.80	13.10	19.10	32.50	11.70	3.13	66.16	0.000
**Identify the blocking effects and possible contradictions with reduction**	29.00	10.70	32.50	20.80	21.30	2.95	31.62	0.000
**Identification of synergy with other policies of the city, estimation of added value**	9.80	9.60	20.80	27.90	33.30	3.65	82.77	0.000

The obtained results regarding the structural resilience of the building under flood conditions for each of the dependencies on environmental ecosystems are presented in Table 4. As shown in the table, considering the single-variable chi-square (χ^2^) values and the significance level smaller than 0.05, there is a statistically significant difference in all studied dependencies between the observed frequencies and the expected frequencies.

**Table 4:** Attitude of dependence on environmental ecosystems.

object	Frequency distribution - percentage					Average	Chi-square value	Significance level (Sig)
	very low	low	moderate	high	very high			
**Flood control**	15.80	18.00	12.30	35.00	18.90	3.23	56.00	0.00
**Bio-climatic design and management**	18.90	2.70	5.70	23.80	48.90	3.81	247.55	0.00

The results obtained regarding the structural resilience of the building under flood conditions for each of the diversity components are outlined in Table 5. As indicated in the table, considering the single-variable chi-square (χ^2^) values and the significance level smaller than 0.05, there is a statistically significant difference in all studied diversity components between the observed frequencies and the expected frequencies.

**Table 5:** Attitude of diversity.

object	Frequency distribution - percentage					Average	Chi-square value	Significance level (Sig)
	very low	low	moderate	high	very high			
**Spatial diversity**	10.90	9.00	14.50	42.10	23.50	3.58	134.14	0.00
**Key assets and tasks that are physically distributed and not all affected by a specific event at any time.**	8.50	9.60	17.20	27.30	37.40	3.67	111.10	0.00
**Functional diversity - multiple ways of dealing with a particular need**	21.90	10.90	17.50	24.30	25.40	3.20	25.61	0.00
**Equilibrium variation with potential cascading effects**	6.80	1.60	4.90	29.00	57.70	4.29	409.16	0.00

The results obtained regarding the structural resilience of the building under flood conditions for each of the learning, memory, and prediction components are presented in Table 6. As indicated in the table, considering the single-variable chi-square (χ^2^) values and the significance level smaller than 0.05, there is a statistically significant difference in all studied components of learning, memory, and prediction between the observed frequencies and the expected frequencies.

**Table 6:** Attitude of learning _ memory _ prediction.

object	Frequency distribution - percentage					Average	Chi-square value	Significance level (Sig)
	very low	low	moderate	high	very high			
**Learning from past experiences and failures**	5.20	15.60	49.20	14.50	15.60	3.20	208.70	0.00
**Use information and experience to create new adaptations**	7.10	16.90	27.90	23.20	24.90	3.42	49.71	0.00
**Avoid repeating past mistakes**	5.50	10.90	10.40	15.80	57.40	4.09	329.46	0.00
**Collect, store, and share experience**	9.60	19.10	14.80	15.60	41.00	3.59	109.27	0.00
**Construction based on long-term value and history of the city**	1.40	4.40	7.70	15.00	71.60	4.51	627.63	0.00
**Integrating resilience into long-term development scenarios**	3.8	9.8	13.7	17.5	55.2	4.1	301.923	0.00

The findings concerning the structural resilience of the building in flood conditions for each performance component are delineated in Table 7. As the table indicates, taking into account the single-variable chi-square (χ^2^) values and the significance level below 0.05, there exists a statistically significant disparity in all examined performance components between the observed and expected frequencies.

**Table 7:** Attitude of performance.

object	Frequency distribution - percentage					Average	Chi-square value	Significance level (Sig)
	very low	low	moderate	high	very high			
**Performance capacity**	7.70	17.20	10.70	32.80	31.70	3.64	100.26	0.00
**System quality**	6.80	17.20	12.80	35.50	27.60	3.60	97.17	0.00
**Self-sufficiency - reducing external dependence**	7.40	19.10	10.90	35.50	27.00	3.56	97.53	0.00
**It performs better than other buildings**	3.00	5.70	7.10	37.40	46.70	4.19	306.79	0.00

The findings pertaining to the building's resilience under flood conditions for each element of response speed are presented in Table 8. As indicated by the table, taking into account the single-variable chi-square (χ^2^) values and the significance level below 0.05, there is a statistically significant distinction in all examined response speed components between the observed and expected frequencies.

**Table 8:** Attitude of response speed.

object	Frequency distribution - percentage					Average	Chi-square value	Significance level (Sig)
	very low	low	moderate	high	very high			
**In taking casualties, including death and illness**	7.40	10.90	21.90	13.70	46.20	3.80	579.18	0.00
**Reorganization**	1.00	4.60	3.80	36.90	51.60	4.30	379.18	0.00
**Maintaining performance and restoring it**	7.10	6.60	19.10	13.40	53.80	4.00	281.02	0.00
**Restore the structure**	3.60	3.80	10.90	18.00	63.70	4.34	462.01	0.00
**Establishing public order**	9.30	4.40	6.60	15.30	64.50	4.21	464.87	0.00
**Prevent future disruption**	6	7.7	10.1	13.9	62.3	4.19	415.721	0.00

The presented Table 9 outlines the results concerning the building's resilience in flood conditions across various incremental segmentation components. Statistical analysis, utilizing the chi-square (χ^2^) test with a significance level below 0.05, reveals a significant difference in observed frequencies compared to expected frequencies for all studied incremental segmentation components.

**Table 9:** Attitude of segmentation redundancy.

object	Frequency distribution - percentage					Average	Chi-square value	Significance level (Sig)
	very low	low	moderate	high	very high			
**Replacement of systems or systems agents**	7.90	9.30	3.00	4.13	66.40	4.21	502.42	0.00
**Buffer from external shocks or changes in demand**	3.00	1.10	6.30	17.80	4.54	4.54	638.70	0.00
**Replacing components with modular parts**	7.70	18.60	36.30	22.70	3.18	3.18	83.48	0.00
**Balance redundancy with potential cascading effects**	7.90	19.10	31.40	25.70	3.22	3.22	59.77	0.00

The results pertaining to the structural resilience of the building under flood conditions for each of the strategic components are detailed in Table 10. As indicated by the table, considering the single-variable chi-square (χ^2^) values and the significance level smaller than 0.05, there is a statistically significant difference in all studied strategic components between the observed frequencies and the expected frequencies.

**Table 10:** Attitude of resourcefulness.

object	Frequency distribution - percentage					Average	Chi-square value	Significance level (Sig)
	very low	low	moderate	high	very high			
**Identifying and predicting problems**	5.70	19.70	23.80	40.20	10.70	3.30	130.23	0.00
**prioritizing**	4.10	28.40	19.70	37.70	10.10	21.30	134.52	0.00
**Resource mobilization, visualization, planning, collaboration and action**	1.40	4.40	7.70	15.00	71.60	4.51	627.63	0.00
**re-evaluation**	3.80	9.80	13.70	27.30	55.20	4.10	301.92	0.00
**Integrating resilience into work processes and administration**	8.5	9.6	17.2	25.4	27.4	3.76	111.104	0.00
**Getting cooperation from citizens**	21.9	10.9	17	24.9	25.4	3.2	25.612	0.00

Table 11 provide the results of the building's resilience under flood conditions for various strategic components. Statistical analysis, using the chi-square (χ^2^) test with a significance level below 0.05, indicates a significant difference in observed frequencies compared to expected frequencies for all studied strategic components.

**Table 11:** Attitude of strength.

object	Frequency distribution - percentage					Average	Chi-square value	Significance level (Sig)
	very low	low	moderate	high	very high			
**Resistance to a level of stress**	3.00	4.60	3.80	36.90	51.90	4.30	379.25	0.00
**Without degradation and loss of performance**	9.30	4.60	8.50	14.80	62.80	4.17	429.38	0.00
**Capacities that guarantee adequate margins**	6.00	7.70	10.10	13.90	62.30	4.19	415.72	0.00

After evaluating the resilience of buildings in floods across various dimensions, the status of each item defined for each dimension and criteria was analyzed using the univariate Chi-square test, acknowledging the non-normality of the data. The results, as indicated by the univariate χ^2 value and the significance level (which is less than 0.05) in all studied items, demonstrate a significant difference between the observed frequency and the expected frequency.

**Fig. 4:**
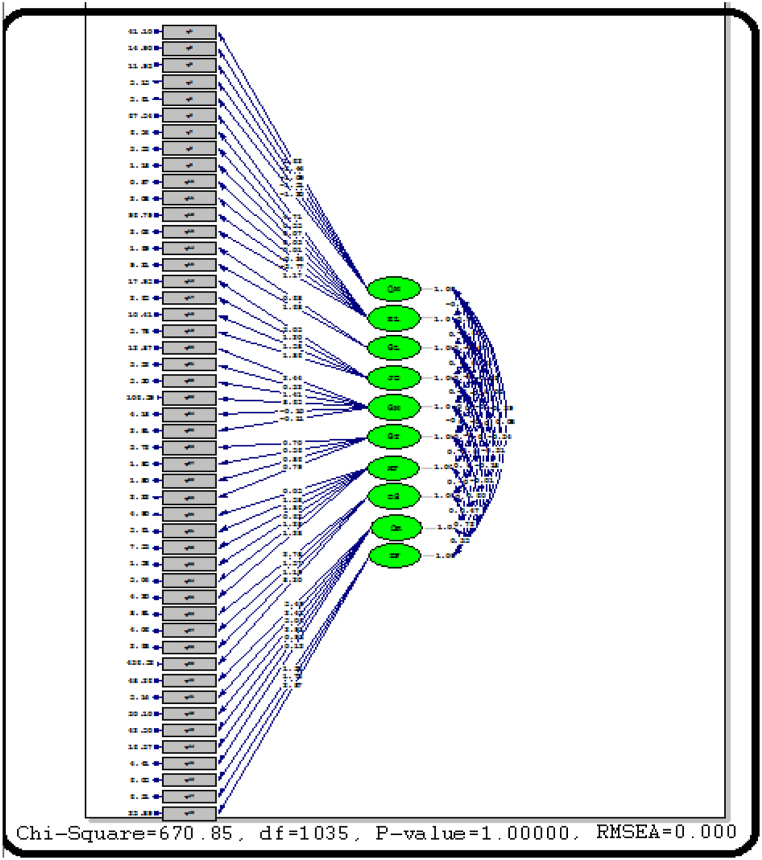
Output of the coefficients of the second-order factor analytical model of building resilience in floods.

Given that the highest frequencies for all items are within the medium, high, and very high classes, it can be concluded that each aspect related to adaptation, flexibility, feedback connection, failure safety, dependence on environmental ecosystems, diversity, learning, memory, prediction, performance, speed of response, redundancy, segmentation, planning, and strength significantly influences building resilience in floods. The fundamental structural model for the building resilience index in floods is illustrated in Fig. 4. The strength of the relationship between latent variables and observable variables is depicted by the factor load, where a factor load less than 0.3 indicates a weak relationship, a factor load between 0.3 and 0.6 signifies an acceptable relationship, and a factor load exceeding 0.6 is considered desirable.

Building resilience in floods comprises 10 micro-criteria. The factor loads for these 10 variables on building resilience in floods are (1.89), (1.56), (0.54), (0.95), (0.87), (0.91), (0.29), (0.43), (0.67), as shown in Fig. 4. All coefficients are deemed acceptable and have been approved.

**Fig. 5:**
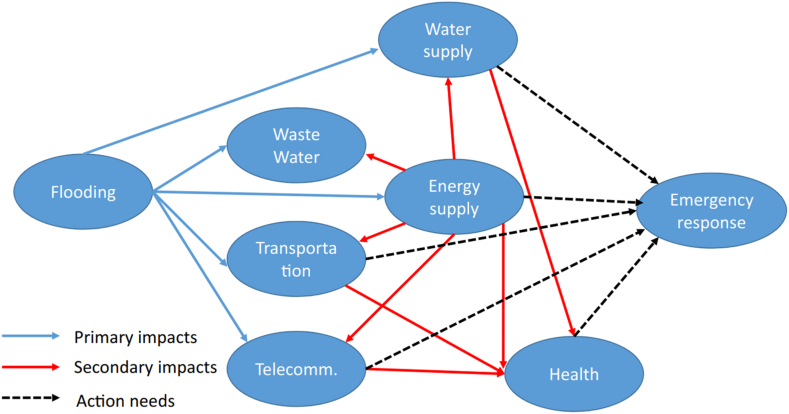
Impacts of flooding to various types of building resilience and interdependencies.

The output of T coefficients for the resilience components of the building in floods, as per Fig. 4, is as follows: 7.23, 6.73, 4.55, 11.73, 4.16, 3.28, 3.78, 5.97, 32, 6.6, and 9.65. All coefficients surpass 2.59 at a significance level of 0.01 (T coefficients between 1.96 and 2.58 at the 0.05 level, and T coefficients higher than 2.85 at the significance level of 0.1 are 0). Fig. 5 shows the building resilience against floods.

The general indicators of the tested building resilience model in floods are presented in Table 12. The goodness of fit index and adjusted index of fit criteria suggest that the model exhibits an average fit. Additionally, the results from Fig. 4 demonstrate the appropriateness of the factor loadings for indicators (questions) related to each component. The factor loading for each component is at a relatively suitable level, indicating the resilience of the building in floods and the predictive capability of this variable.

**Table 12:** General fit indices of the tested model of building resilience in floods.

characteristic	estimate	Criterion
**The ratio of chi square to degrees of freedom**	0.002	df > 3x∗2
**root mean square error estimate (RMSEA)**	0.0009	0/08> RMSEA>0
**goodness of fit index (GFI)**	0.79	1> GFI>0.9
**Adjusted goodness of fit index (AGFI)**	0.75	1> AGFI>0.9
**Comparative Fit Index (CFI)**	0.87	1> CFI>0.9
**Softness of Fit Index (NFI)**	0.63	1> NFI>0.9

As presented in Table 13, the goodness of fit index for the building resilience model in floods is calculated as 0.79. According to the estimation criteria for the goodness of fit index, any value closer to one indicates a good model fit. The adjusted goodness of fit index was utilized to account for the model's complexity. The adjusted goodness of fit index adjusts the goodness of fit index based on the relationship between the sample size and the degrees of freedom of the model. Each construct is evaluated at a relatively suitable level. The average root square of the approximation error for all sub-components of the building resilience model in floods is 0.07. The comparative fit index is 0.87, and the Tucker-Lewis Index is 0.63. While closer to one indicates more acceptability, the model fit is relatively average but good.

**Table 13:** Pearson correlation matrix of variables.

Variable	1	2	3	4	5	6	7	8	9	10
**Adaptability - flexibility**	1									
**Fail safe feedback connection**	0.861	1								
**Dependence on environmental ecosystems**	0.624	0.731	1							
**Variety**	0.623	0.703	0.719	1						
**Learning _ memory _ prediction**	0.502	0.471	0.251	0.387	1					
**Function**	0.648	0.617	0.423	0.46	0.604	1				
**Response speed**	0.245	0.236	0.153	0.1	0.213	0.242	1			
**Redundancy of segmentation**	0.103	0.122	0.114	0.089	0.065	0.1	0.611	1		
**resourcefulness**	0.323	0.198	0.187	0.101	0.231	0.342	0.231	0.245	1	
**Strength**	0.201	0.312	0.167	0.232	0.187	0.197	0.311	0.341	0.237	1

According to Table 14, significant correlation coefficients indicate the strength of the relationship between variables. A coefficient in the range of (0.3) suggests a weak relationship, while (0.6) implies a moderate relationship, and (0.6, 0.1) indicates a strong relationship. It is important to note that the direction of the relationship is determined by the sign of the correlation coefficient. A positive correlation coefficient signifies a direct and positive relationship, while a negative coefficient indicates an inverse and negative relationship between the two variables. In this section, the research findings are analyzed based on the research questions, aiming to draw conclusions about the characteristics of the society from which the studied sample was drawn. Confirmatory factor analysis and structural equation modeling have been employed to analyze research questions and hypotheses.

**Table 14:** Results of structural equation modeling analysis.

Standard coefficient	T - Value	Predictor variable	Criterion variable	Test result
**0.95**	7.23	Adaptability - flexibility	Resilience of building in floods	H1
**0.97**	6.73	Fail safe feedback connection	Resilience of building in floods	H1
**0.83**	4.55	Dependence on environmental ecosystems	Resilience of building in floods	H1
**0.74**	11.73	Variety	Resilience of building in floods	H1
**0.77**	4.16	Learning _ memory _ prediction	Resilience of building in floods	H1
**0.53**	3.28	Function	Resilience of building in floods	H1
**0.24**	3.78	Response speed	Resilience of building in floods	H1
**0.14**	5.97	Redundancy of segmentation	Resilience of building in floods	H1
**0.65**	6.32	resourcefulness	Resilience of building in floods	H1
**0.95**	9.65	Strength	Resilience of building in floods	H1

The variables encompassed within the components collectively contribute to shaping the resilience of buildings in the face of floods. These elements represent critical factors that influence a building's capacity to adapt, respond, and endure the challenges posed by flood events.

$$H_{0}:B_{i}=0\;H_{0}:B_{i}\neq 0$$

H_0_: The assertion that the components (adaptability-flexibility, failure-safe feedback connection, dependence on environmental ecosystems, diversity, learning-memory-prediction, performance, response speed, segmentation redundancy, resourcefulness, strength) do not function as variables affecting building resilience in floods.

H_1_: The hypothesis suggesting that the criteria act as variables influencing building resilience in floods. In the examination of this hypothesis through a structural equation model, the software output affirms the appropriateness of the fitted structural model for assessing the research hypotheses.

In this study, the Expert Choice11 software was employed to determine the inconsistency rate, yielding a value of 0.06. This indicates an acceptable level of consistency in the pairwise comparisons of the criteria, as illustrated. The criteria of balance redundancy with a weight of 0.077 and performance capacity with a weight of 0.056, along with the replacement index of systems or system factors with a weight of 0.054, hold the highest weights. Conversely, the indicators related to learning from past experiences, failures, and utilizing information and experience to foster new adaptation and resilience exhibit the lowest weight, standing at 0.003. Consequently, prioritizing enhancements in balance redundancy and increasing performance capacity emerges as crucial steps in building resilience programs to mitigate the impact of floods.

## 4 Conclusion

This comprehensive study addresses the critical issue of urban resilience against flood threats, emphasizing the importance of recognizing and enhancing the resilience of essential buildings in key urban centers. The research employs a systematic approach, incorporating GIS technology for spatial mapping, HEC-RAS software for river flow modeling, and advanced methodologies like TOPSIS and AHP for resilience modeling. The identification and prioritization of city assets, including physical, human resource, information exchange, and spiritual assets, lay the foundation for subsequent analyses. The integration of asset adaptation and hydraulic modeling of rivers within the GIS platform reveals vulnerable focal points in Hamadan city. The determination of building resilience components, using the Resilience Index and LISREL modeling software, results in a robust model comprising ten key variables influencing resilience against floods. The application of the combined AHP-TOPSIS method for ranking resilience indicators further refines the assessment. The results and analysis section underscores the significance of adaptability, flexibility, feedback connection, environmental ecosystems, diversity, learning, memory, prediction, performance, response speed, redundancy, segmentation, planning, and strength in influencing building resilience against floods. The structural equation modeling confirms the validity of the identified resilience components, and the goodness of fit indices indicate an average to good fit of the model. Furthermore, the study identifies priorities for building resilience programs, emphasizing the importance of balance redundancy and performance capacity in mitigating the impact of floods. The use of Expert Choice software validates the consistency of criteria and highlights key areas for improvement in building resilience.The findings from this research contribute valuable insights and a comprehensive model for understanding, assessing, and enhancing building resilience in the face of flood threats. These outcomes can guide urban planners, policymakers, and stakeholders in implementing effective strategies to safeguard critical infrastructure and improve overall urban resilience.

## CRediT authorship contribution statement

**Faraz Estelaji:** Writing – review & editing, Writing – original draft. **Rahim Zahedi:** Supervision, Software. **Arash Gitifar:** Methodology, Investigation. **Alireza Naseri:** Visualization, Validation. **Mohammad Hossein Yari:** Data curation, Conceptualization. **Bita Rouhi Asl:** Funding acquisition, Formal analysis. **Bita Abedi:** Resources, Project administration.

## Data availability

Datasets analyzed during the current study are available and can be given following a reasonable request from the corresponding author.

## Declaration of competing interest

The authors declare that they have no known competing financial interests or personal relationships that could have appeared to influence the work reported in this paper.
